# Effectiveness comparisons of various therapies for FIGO stage IB2/IIA2 cervical cancer: a Bayesian network meta-analysis

**DOI:** 10.1186/s12885-021-08685-9

**Published:** 2021-10-06

**Authors:** Jing Cheng, Beibei Liu, Biao Wang, Xicui Long, Zhihong Li, Ruili Chen, Ruiting Wu, Lin Xu

**Affiliations:** 1 Department of Obstetrics and Gynecology, The First Affiliated Hospital of Kunming Medical University, No. 295, Xichang Road, Wuhua District, Kunming City, 650000 Yunnan Province China; 2 Department of Urology, The First Affiliated Hospital of Kunming Medical University, No. 295, Xichang Road, Wuhua District, Kunming City, 650000 Yunnan Province China; 3 Department of Gynecology, Kunming Tongren Hospital, No. 1099 Guangfu Road, Xishan District, Kunming City, 650100 Yunnan Province China

**Keywords:** Cervical cancer, Concomitant chemoradiotherapy, Surgery, Neoadjuvant chemotherapy, Radiotherapy, Bayesian network meta-analysis

## Abstract

**Background:**

Cervical cancer is a common malignancy of the female genital tract. Treatment options for cervical cancer patients diagnosed at FIGO (2009) stage IB2 and IIA2 remains controversial.

**Methods:**

We perform a Bayesian network meta-analysis to directly or indirectly compare various interventions for FIGO (2009) IB2 and IIA2 disease, in order to improve our understand of the optimal treatment strategy for these women. Three databases were searched for articles published between 1971 and 2020. Data on included study characteristics, outcomes, and risk of bias were abstracted by two reviewers.

**Results:**

Seven thousand four hundred eighty-six articles were identified. Thirteen randomized controlled trials of FIGO (2009) IB2 and IIA2 cervical cancer patients were included in the final analysis. These trials used six different interventions: concomitant chemoradiotherapy (CCRT), radical surgery (RS), radical surgery following chemoradiotherapy (CCRT+RS), neoadjuvant chemotherapy followed by radical surgery (NACT+RS), adjuvant radiotherapy followed by Radical surgery (RT + RS), radiotherapy alone (RT).SUCRA ranking of OS and Relapse identified CCRT+RS and CCRT as the best interventions, respectively. Systematic clustering analysis identified the CCRT group as a unique cluster.

**Conclusion:**

These data suggest that CCRT may be the best approach for improving the clinical outcome of cervical cancer patients diagnosed at FIGO (2009) stage IB2/IIA2. Phase III randomized trials should be performed in order to robustly assess the relative efficacy of available treatment strategies in this disease context.

**Supplementary Information:**

The online version contains supplementary material available at 10.1186/s12885-021-08685-9.

## Introduction

Cervical cancer is a major cause of morbidity and mortality, and remains one of the four most common malignant tumors in women. Globally, more than 560,000 new cases of cervical cancer are diagnosed each year, of which 80% occur in developing countries [1, 2].

Treatments of stage IB2/IIA2 cervical cancer revolves around chemoradiotherapy (CCRT), radical Surgery (RS), radical surgery following chemoradiotherapy (CCRT+RS), neoadjuvant chemotherapy followed by radical surgery (NACT+RS), adjuvant radiotherapy followed by Radical surgery (RT + RS), radiotherapy alone (RT). Previous studies have suggested that CCRT is the most appropriate treatment strategy [3–6]. However, other investigators have reported that NACT + RS improves the long-term DFS and OS of patients with locally advanced disease [7–9]. Other treatment regimens, such as CCRT+RS [10, 11], RT + RS [11, 12], RT [13, 14] and RS [14, 15], remain controversial. We therefore sough to perform a network meta-analysis of currently available findings in order to determine the most effective treatment for patients with stage IB2/IIA2 cervical cancer.

Systematic reviews and meta-analyses are widely considered to represent the pinnacle of the medical evidence pyramid [16]. However, traditional meta-analysis typically compare only two intervention types. In contrast, network meta-analyses can process all possible comparison indicators in the same model multiple times or in combination, and collect direct and indirect evidence at the same time [17, 18]. Moreover, network meta-analyses are thought to produce more accurate and reliable models compared to traditional meta-analysis, representing the premier guideline evidence for clinical practice [19, 20]. A network meta-analysis compares multiple treatment options for the same disease, which may be useful for developing clinical practice guidelines [21].

Here, we present a Bayesian network meta-analysis to address the currently conflicting data surrounding optimal treatment strategies for FIGO IB2/IIA2 cervical cancer patients. We aim to summarize and analyze the existing evidence to explore the clinical outcome of patients treatment with various regimens, using overall survival (OS) and disease recurrence as primary endpoints, in order to identify the optimal approach for management of locally advanced disease.

## Material and methods

### Search strategy and study selection

Two authors performed independent searches using PubMed, the Cochrane Central Register of Controlled Trials and Embase to identify Randomized Controlled Trials (RCTs) for the treatment of cervical cancer from 1971 to 2020, according to the Cochrane System Intervention Review Manual [22]. A comprehensive search was carried out through Boolean logic operators with Medical Subject Headings (MeSH) combined with entry words, using “Uterine Cervical Neoplasms”, “Chemoradiotherapy”, “General Surgery”, “Surgical Procedures, Operative”, “Gynecologic Surgical Procedures”, “Hysterectomy”, “Chemotherapy, Adjuvant”, “Drug Therapy”, “Radiotherapy” and “Randomized controlled trials”. This study was conducted based on the Preferred Reporting Items for Systematic Reviews and Meta-Analyses (PRISMA) for systematic reviews and meta-analysis [23] (Material S2). The specific search strategy is detailed in Material S1.

As specified in the predetermined inclusion criteria, all searched articles were individually evaluated by the two authors. We first screened the initial inclusion of studies based on the title and abstract, and deleted duplicate studies. Remaining articles were subject to full text screening by the two authors to evaluate study relevance. All citations were managed in Endnote X9. In order to ensure that further analysis can proceed smoothly, it is necessary to check the veracity and completeness of the data. Discrepancies between the two authors were resolved by a third empirical observer through discussion.

### Inclusion and exclusion criteria, data extraction

The two authors independently extracted relevant data for each included trial. Discrepancies were addressed via discussion and consensus, with external arbitration where required.

Detailed inclusion and exclusion criteria are shown in Table S1. In our inclusion and exclusion criteria, treatment is defined as a preference. Treatments were defined as an intervention following discussion of the physician and patient, including surgery, radiotherapy, chemotherapy, or a combination of these regimens. All included randomized controlled trials were coded according to treatment type and are divided into 6 treatment groups. Differences in coding between the two authors were resolved by discussion and consensus, with external arbitration where required.

### Quality appraisal, evaluation of endpoints

We used Cochrane tools to assess the risk of bias (ROB) of the included studies [22]. The two authors separately assessed seven areas of ROB. ROB evaluation is conducted in Review Manager (version 5.1).

The primary endpoints were overall survival and disease relapse; comparisons of all interventions were performed. All surviving patients contribute to OS, regardless of their disease status. Where exact case numbers of deceased and surviving patients were not available, these were estimated from Kaplan-Meir survival curves; corresponding authors of included studied were contacted where necessary. Both local recurrence and distant metastasis were included as disease relapse.

### Statistical analysis

Compared with traditional meta-analysis, Bayesian network meta-analysis has greater analytical power, in that it summarizes all possible intervention comparisons simultaneously [20]. Using the minimum information prior distribution based on the random effect Bayesian statistical model, a connection network is formed combining direct and indirect evidence. Six intervention therapies were compared simultaneously; first, we performed regular pairwise meta-analysis. The I^2^ index was used to determine heterogeneity; indices of 25, 50, and 75% represent mild heterogeneity, moderate heterogeneity, and high heterogeneity, respectively [24]. A funnel chart was produced to detect publication bias. In order to reveal all available treatment evidence, a simple summary description network diagram was generated. The above analysis was performed in STATA, version 14.2. Endpoint analysis effect sizes were summarized as odds ratios (OR) with corresponding confidence intervals (CrI). Bayesian stratified random effects were used to directly and indirectly compare multiple interventions. The Bayesian method is used to calculate endpoint results; first, three parallel Markov chains with randomly selected states are established to simulate accurate estimation of statistical models [25]. Each chain generates 50,000 iterations, and because of the aging cycle, the first 20,000 iterations will be abandoned to ensure minimization of deviation of the initial value [26]. Convergence of the model was judged through the diagnostic curve [27]. The surface under the Cumulative Ranking Curve (SUCRA) is regarded as the ranking probability map for each intervention. The higher the SUCRA value, the more likely it is that an intervention is at the highest level or very effective, while a value of 0 means that the treatment is least effective [28]. Consistency between the two comparisons was evaluated by comparing the DIC values between the consistency and inconsistency models (a difference greater than 5 is considered as inconsistency between models) [29]. Node splitting was used to further assess for local inconsistencies in our network [30]. These analyses were performed using R (X64 version 3.5.3) with the “Gemtc” package (0.8–4 version), “JAGS” (version 4.3.0) and OpenBUGS (version 3.2.3).

### Cluster analysis of the treatments

After Bayesian network analysis, by sorting out the SUCRA data of OS and relapse, a systematic cluster analysis of various treatment options was performed. Two to five cluster types were chosen and a vertical icicle diagram was used to visualize different clustering forms. After the systematic clustering analysis, the results were further analyzed through Online Analytical Processing (OLAP). The above analysis uses IBM SPSS version 26.0 for analysis.

## Results

### Study characteristics and ROB quality assessment

**Fig. 1:**
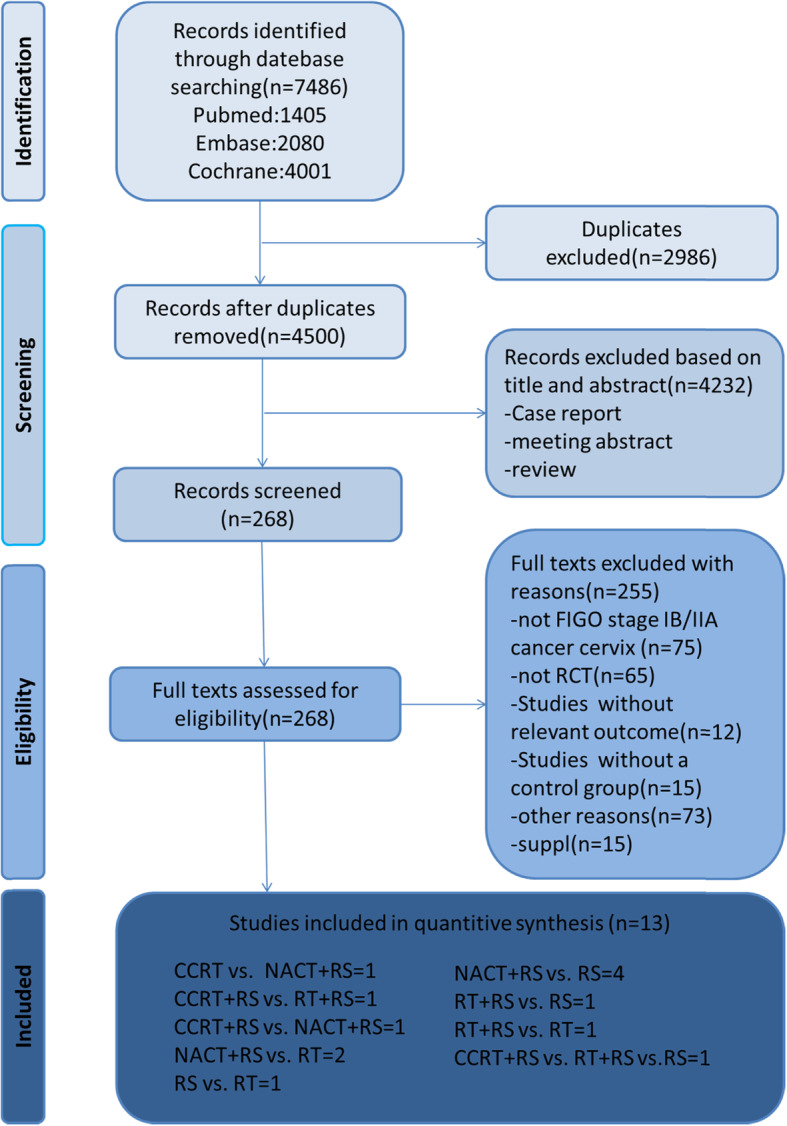
Literature review flow chart. Note: RCT = Randomized controlled trial, FIGO=International Federation of Gynecology and Obstetrics, RS = Radical Surgery, CCRT = concomitant chemotherapy and radiotherapy, NACT = neoadjuvant chemotherapy, RT = radiotherapy

Among the 7486 citations, 4500 records were retained after deletion of duplicates. Four thousand two hundred thirty-two citations were removed after evaluation of title and abstract. Two hundred fifty-five records were excluded during full-text screening: 75 studies did not include stage IB2 and IIA2 cervical cancer, 65 studies were not randomized controlled trials, 12 studies had no relevant results, 15 studies did not determine the control group, 15 studies were supplements and 73 were excluded for other reasons such as foreign language, abstract etc.13 articles were included in the final study (Fig. 1).

These studies included 2733 participants undergoing 6 different interventions and provided sufficient data published from 1987 to 2020. Table 1 summarizes the main characteristics of the participants and interventions in the 13 included studies. Overall, 1399 patients were randomly assigned to the intervention group, while the remaining 1334 patients were assigned to the control group. In different studies, age is reported as the mean or median, ranging from 18 to 70 years. Across the 13 randomized controlled trials, most (more than half) of the participants were from Asia, followed by North America and Europe.

**Table 1:** Characteristics of included studies

Study	Country	RCT	FIGO stage	Follw up,m	Age(years)	Outcomes		ROB
						OS	Recurrence	
Gupta 2018 [31]	India	Y	IB2,IIA,IIB	58.5	18–65	Y	Y	L
Li 2010 [32]	China	Y	IB2-IIB	120	NR	Y	Y	H
Curtin 1996 [33]	American	Y	IB-IIA	60	45(23–70)		Y	L
Peters 2000 [11]	American	Y	IA2,IB,IIA	60	NR	Y		L
Benedetti 2002 [34]	Italy	Y	IB2-III	24	less than 70	Y		L
Chang 2000 [35]	Taiwan	Y	IB,IIA	39	46(33–69), 47(32–70)	Y	Y	L
Wang 2020 [36]	China	Y	IB2-IIB	36	more than 20		Y	L
Chen 2008 [37]	China	Y	IB2-IIB	48	44(25–74)	Y	Y	L
Duan 2017 [38]	China	Y	IB,IIA	NR	27–66,29–67		Y	L
Katsumata 2013 [39]	Japan	Y	IB2,IIA2,IIB	42	20–70	Y		L
Landoni 2017 [13]	Italy	Y	IB-IIA	228	NR	Y	Y	L
Li 2008 [12]	China	Y	IB2,IIA	30	25–75	Y	Y	L
Perez 1987 [40]	Mexico	Y	IB,IIA	60	less than 70		Y	L
Study	Inventions and Sample size				Intervention details			
Gupta 2018 [31]	CCRT = 317 vs NACT+RS = 316				NACT+RS group: Paclitaxel combined with carboplatin was taken every three weeks for three cycles, and then a total hysterectomy was performed. CCRT group: standard radiotherapy combined with cisplatin once a week for 5 weeks.			
Li 2010 [32]	CCRT+RS = 64 vs RT + RS = 73 vs RS = 122				CCRT + RS group: a total of 2 to 3 times, each dose of 1 week interval is 600–1000 cGy, and the total dose is 2000–300 cGy. The chemotherapy regimen is 5-FU 3.5–4.0 g/m2, continuous injection with a micropump for 96 h. DDP is 70 mg/m2, and intravenous chemotherapy is given for 1–2 days. RT + RS group: radiotherapy after intracavitary loading before surgery. RS group: radical resection of cervical cancer.			
Curtin 1996 [33]	CCRT+RS = 44vs NACT+RS = 45				CCRT + RS group: 2 cycles of chemotherapy with an interval of 3–4 weeks, using bleomycin 20 U/m2 every day on Days 1–3. On the 4th day, 75 mg/m2 of cisplatin was infused intravenously. The radiation dose is 45 Gy.RS: Radical hysterectomy and pelvic lymph node dissection. NACT + RS group: After the first two cycles of cisplatin and bleomycin treatment as above, the patient subsequently received two separate cisplatin treatments.RS: Radical hysterectomy and pelvic lymph node dissection.			
Peters 2000 [11]	CCRT+RS = 127 vs RS + RT = 116				CCRT + RS group: The radiation dose was 49.3 GY. The chemotherapy regimen included 4 cycles of 70 mg/m^2^ cisplatin and 1000 mg/m^2^ continuous fluorouracil. RS: radical hysterectomy and pelvic lymph node dissection. RS + RT group: radical hysterectomy and pelvic lymph node dissection plus 49.3 GY radiotherapy.			
Benedetti 2002 [34]	NACT+RS = 210 vs RT = 199				NACT + RS group: cisplatin-based, followed by type III-V radical hysterectomy plus systemic pelvic lymphadenectomy. RT group: external beam radiation therapy (45 to 50 Gy), followed by brachytherapy (20 to 30 Gy).			
Chang 2000 [35]	NACT+RS = 68 vs RT = 52				NACT+RS group included either cisplatin 50 mg/m2 and vincristine 1 mg/m2 for 1 day and bleomycin 25 mg/m2 for 3 days for three cycles followed by radical hysterectomy. RT group received primary pelvic radiotherapy only.			
Wang 2020 [36]	NACT+RS = 60 vs RS = 60				NACT + RS group: TP regimen: Cisplatin (70–80 mg/m2) plus paclitaxel (150–175 mg/m2), TC regimen: carboplatin (AUC = 5) + paclitaxel (150–175)) (mg / m^2^) and TN program: nedaplatin (70–80 mg/m^2^) + paclitaxel (150–175 mg/m^2^). 1–3 cycles of treatment every 3 weeks. Then, perform total hysterectomy and pelvic lymph node dissection. RS group: radical hysterectomy and pelvic lymph node dissection were performed.			
Chen 2008 [37]	NACT+RS = 72 vs RS = 70				NACT + RS group: cisplatin 100 mg/m2 was given intravenously on day 1, mitomycin C 4 mg/m2 and 5-fluorouracil 24 mg/m2 were given from day 1 to day 5. There are two cycles of treatment with an interval of 14 days. After one week of treatment, the patient underwent type III radical hysterectomy and pelvic lymphadenectomy. RS group: The patients directly underwent radical surgery.			
Duan 2017 [38]	NACT+RS = 32 vs RS = 32				NACT + RS group: 200 mg/m2 paclitaxel combined with 50 mg/m2 cisplatin treatment for 2 cycles. Three weeks after the chemotherapy, a radical resection of cervical cancer was performed. RS group: patients only received radical surgery for cervical cancer.			
Katsumata 2013 [39]	NACT+RS = 64 vs RS = 67				NACT + RS group: BOMP regimen (Bleomycin 7 mg/m2 on day 1 to 5, vincristine 0.7 mg/m2 on day 5, Mitomycin 7 mg/m2 on day 5, cisplatin 14 mg/m2 from1 to 5 days, 2 to 4 cycles every 3 weeks) plus type III or type IV radical hysterectomy. RS group: type III or type IV radical hysterectomy alone.			
Landoni 2017 [13]	RS = 172 vs RT = 171				RT group: The median total radiation dose at point A was 76 Gy (range 70–90 Gy). RS group: radical hysterectomy plus pelvic lymphadenectomy extended to level 2.			
Li 2008 [12]	RT + RS = 38 vs RS = 40				RT + RS group: preoperative intracavitary brachytherapy with a dose of 2000–3000 cGy 192Ir. After 10–14 days, a radical hysterectomy combined with pelvic lymph node dissection was performed. RS group: The patients directly received radical surgery.			
Perez 1987 [40]	RS + RT = 62 vs RT = 56				RT group: The radiation dose was 1000 cGy for the whole pelvis, and parametria was used for additional 4000 cGy; RT + RS group: 2000 cGy was used to irradiate the entire pelvis, and then radical hysterectomy and pelvic lymph node dissection were performed 2 to 6 weeks later.			
Study	Conclusion							
Gupta 2018 [31]	Cisplatin-based concomitant chemoradiation resulted in superior DFS compared with neoadjuvant chemotherapy followed by radical surgery in locally advanced cervical cancer.							
Li 2010 [32]	The patients with locally advanced cervical cancer treated with preopera-tive concurrent chemoradiotherapy had more reduction in tumor size than those who did not receive such treatment. Pre-operative concurrent chemoradiotherapy can be considered safe, feasible, and worthy of further study.							
Curtin 1996 [33]	CT + RT did not prove a superior adjuvant therapy for patients at high risk of recurrence after RH-PLND for early cervical cancer in this limited trial. Recurrence rates and patterns of recurrences (local, regional, or distant) were not influenced by the addition of RT.							
Peters 2000 [11]	The addition of concurrent cisplatin-based CT to RT significantly improves progression-free and overall survival for high-risk, early-stage patients who undergo radical hysterectomy and pelvic lymphadenectomy for carcinoma of the cervix.							
Benedetti 2002 [34]	Although significant only for the stageIB2 to llB group, a survival benefit seems to be associ-ated with the NACT+RS compared with conventional RT.							
Chang 2000 [35]	NAC followed by radical hysterectomy and primary R/T showed similar efficacy for bulky stage IB or IIA cervical cancer.							
Wang 2020 [36]	Neoadjuvant chemotherapy can effectivelylower the levels of serum tumor markers and NLR, reducethe metastasis rate of cancer cells and the degree of cancer-related fatigue after operation,improve the quality of lifeand prolong the survival time.							
Chen 2008 [37]	The modified preoperative NAC is well tolerated and beneficial in reducing tumor size, eliminating pathological risk factors, and improving prognosis for responders. It also avoids the delay of effective treatment for non-NAC responders.							
Duan 2017 [38]	Neoadjuvant chemotherapy combined with cervical cancer radical surgery show goodclinical efficacy for treating cervical cancer, and because of the low incidence rate of complications,ithas clinical application value.							
Katsumata 2013 [39]	Neoadjuvant chemotherapy with BOMP regimen before RS did not improve overall survival, but reduced the numberof patients who received postoperative RT.							
Landoni 2017 [13]	The results of the present study seem to suggest that there is no treatment of choice for early stage cervical carcinoma in terms of survival. Long term follow-up confirms that the best treatment for the individual patient should take into account clinical factors such as menopausal status, comorbidities, histological type, and tumor diameter.							
Li 2008 [12]	Preoperativeintracavitary brachytherapy is an effective procedure for the treatment for stage l b. and ll a cervical cancerand can significantly improve the locoregional control rate.							
Perez 1987 [40]	The present study shows no significant difference in therapeutic results or morbidity for invasive carcinoma of theuterine cervix Stage IB or lIA treated with irradiation alone or combined with a radicalhysterectomy and lymphadenectomy.							

*RCT* Randomized controlled trial, *FIGO* International Federation of Gynecology and Obstetrics, *OS* overall survival, *RS* Radical Surgery, *CCRT* concomitant chemotherapy and radiotherapy, *NACT* neoadjuvant chemotherapy, *RT* radiotherapy, *NR* not report, *ROB* risk of bias, *L* low risk, *H* high risk

The 13 included studies included 6 interventions. The number of events and the The quality of individual and overall research levels are plotted in Figure S1 and Figure S2, respectively. In all 13 trials, all sequences were randomly generated, nine randomized controlled trials described their allocation concealment method, one trial design was not double-blind, and four randomized controlled trials had incomplete data on outcome indicators. There is 1 randomized controlled trial with higher risk, which originated from allocation concealment and double-blind design.

I^2^ analysis indicated no statistically significant heterogeneity in our preliminary meta-analysis (I^2^ = 0 for OS, *P* > 0.05, I^2^ = 8% for relapse, *P* > 0.05). The funnel chart indicated no obvious publication bias for OS (Figure S3) or relapse (Figure S4).

**Fig. 2:**
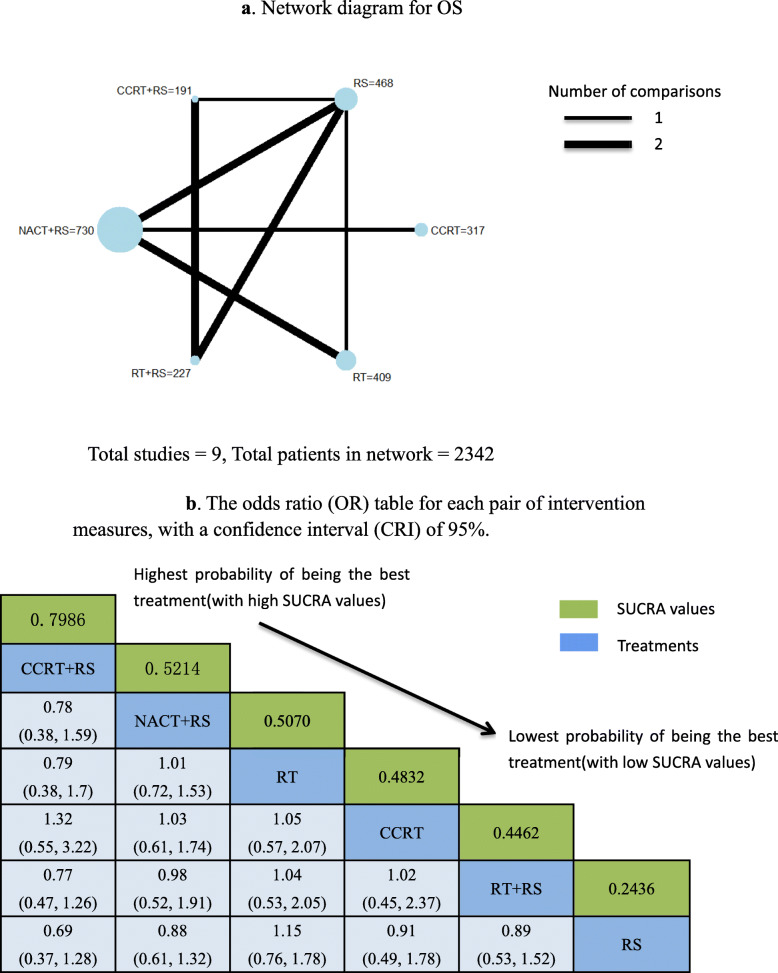
**a** Network diagram for OS. Total studies = 9, Total patients in network = 2342. **b** The odds ratio (OR) table for each pair of intervention measures, with a confidence interval (CRI) of 95%. Odds ratio for OS: Treatment in top left is better. Abbreviations:CCRT = concomitant chemotherapy and radiotherapy,RS = Radical Surgery,NACT = neoadjuvant chemotherapy,RT = radiotherapy

**Fig. 3:**
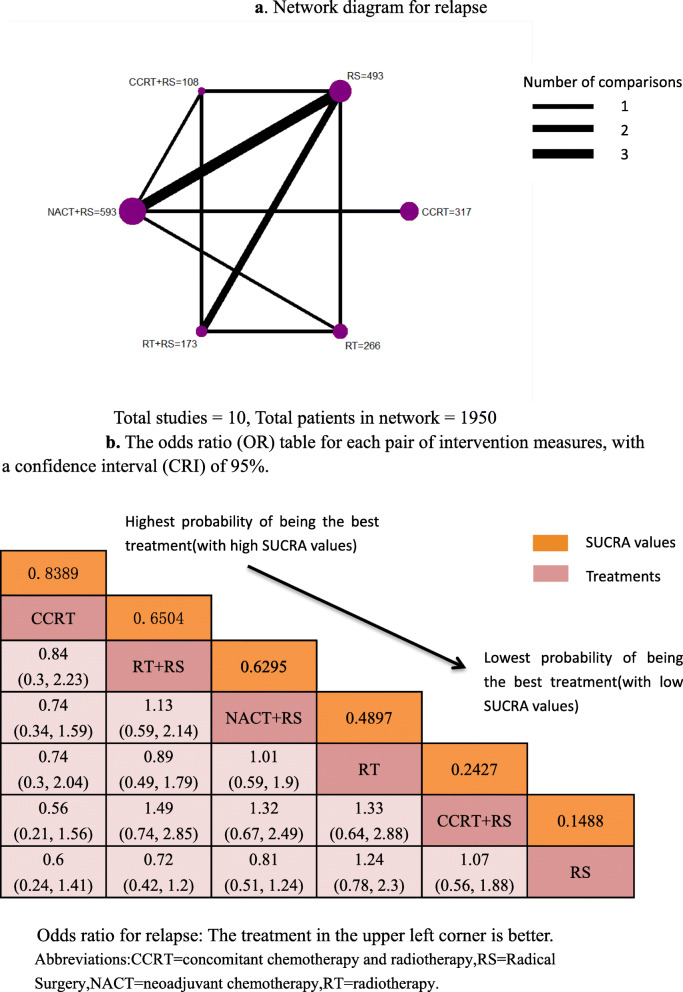
**a** Network diagram for relapse. Total studies = 10, Total patients in network = 1950. **b** The odds ratio (OR) table for each pair of intervention measures, with a confidence interval (CRI) of 95%. Odds ratio for relapse: The treatment in the upper left corner is better. Abbreviations:CCRT = concomitant chemotherapy and radiotherapy,RS = Radical Surgery,NACT = neoadjuvant chemotherapy,RT = radiotherapy

Visual network geometry was performed to show each arm. Each intervention has its own unique nodes, whose size depends on their number in the entire network. The two interventions are connected by straight lines, and the thickness of each straight line represents the number of comparisons (Fig. 2a, Fig. 3a).

Among the 2733 patients, the final number of OS and relapses were 1692 in 2342 and 470 in 1950, respectively (Table 2). A SUCRA line was drawn to rank the hierarchy of each interventions (shown in Fig. 2b and Fig. S5 for OS), which indicated that CCRT+RS got the highest probability (SUCRA = 0.7986) in IB2/IIA2 patients compared with the other 5 active interventions, Following by NACT+RS (SUCRA = 0.5214), RT (SUCRA = 0.5070), CCRT (SUCRA = 0.4832), RT + RS (SUCRA = 0.4462), RS (SUCRA = 0.2436) got an inferior ranking. Another SUCRA line was drawn to rank the hierarchy of each interventions (shown in Fig. 3b and Figure S6 for relapse), which indicated that CCRT got the highest probability (SUCRA = 0.8389) in IB2/IIA2 patients compared with the other 5 active interventions, Following by RT + RS (SUCRA = 0.6504), NACT+RS (SUCRA = 0.6295), RT (SUCRA = 0.4897), CCRT+RS (SUCRA = 0.2427), RS (SUCRA = 0.1488) got an inferior ranking.

**Table 2:** Intervention characteristics of trials for each evaluation endpoint in the network meta-analysis

Treatment	Overall survival			Relapse		
	Trials	^a^Events/Patients	%	Trials	^b^Events/Patients	%
CCRT	1	237/317	74.8	1	43/317	13.6
RS	7	338/468	72.2	6	175/493	35.5
CCRT+RS	3	156/191	81.7	2	37/108	34.3
NACT+RS	8	512/730	70.1	6	106/593	17.9
RT + RS	4	173/227	76.2	3	41/173	23.7
RT	4	276/409	67.5	3	68/266	25.6
**Total**		1692/2342	72.2		470/1950	24.1

*Abbreviations*: *CCRT* concomitant chemotherapy and radiotherapy, *RS* Radical Surgery, *NACT* neoadjuvant chemotherapy, *RT* radiotherapy. ^a^People who were alive during follow-up. ^b^Patients with local or distant metastasis

### Inconsistency detection

The posterior values of the random effects inconsistency and consistency model were estimated; for OS and relapse, the difference in DIC values between the consistency and inconsistency model was 2.6 and 2.0, respectively. These indicated no substantial inconsistency between models.

### Overall ranking of SUCRA for each endpoint and cluster analysis

**Fig. 4:**
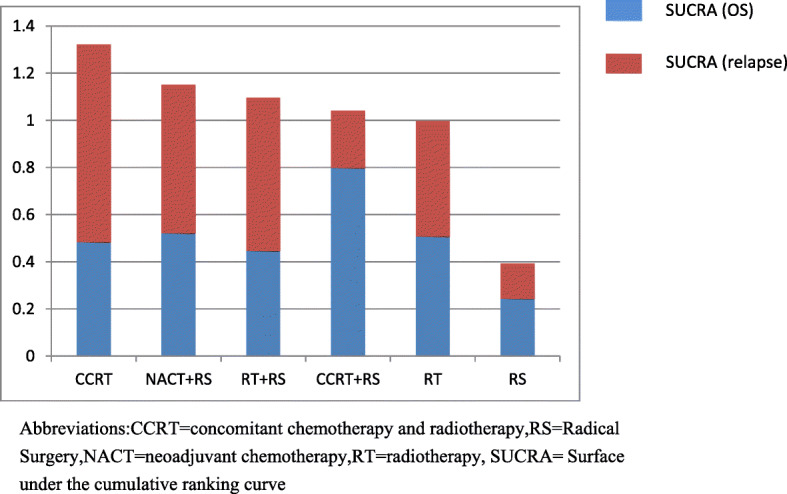
SUCRA ranking of all endpoint. Abbreviations: CCRT = concomitant chemotherapy and radiotherapy, RS = Radical Surgery, NACT = neoadjuvant chemotherapy, RT = radiotherapy, SUCRA = Surface under the cumulative ranking curve

**Fig. 5:**
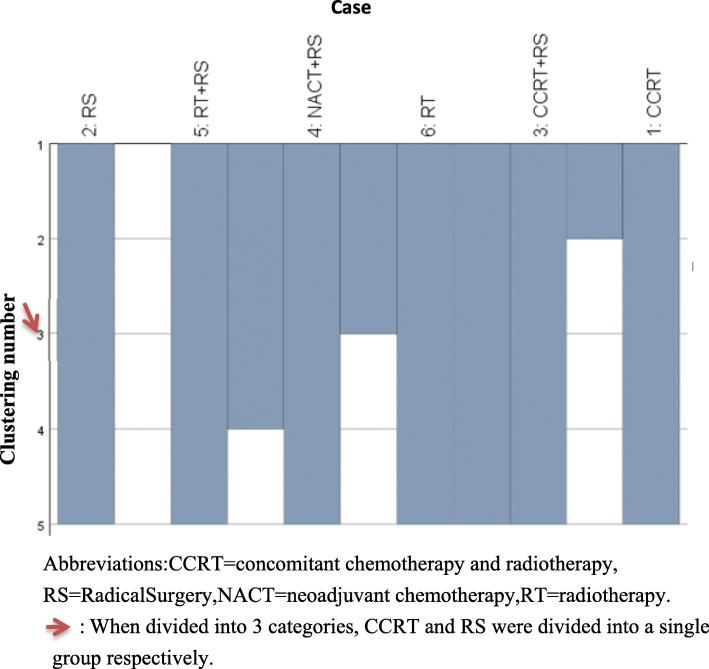
Hierarchical clustered icicle diagram. Abbreviations:CCRT = concomitant chemotherapy and radiotherapy, RS = RadicalSurgery,NACT = neoadjuvant chemotherapy,RT = radiotherapy.  : When divided into 3 categories, CCRT and RS were divided into a single group respectively

Intervention ranking were distinct for the two endpoints measures (OS and relapse). Clinically, high OS is highly desirable; however, high recurrence rate also represent a substantial burden on patients. In order to make an overall assessment of the best treatment plan, the SUCRA value of each endpoint of all 13 interventions was added to obtain a cumulative SUCRA score. This analysis determined CCRT as the optimal treatment strategy (Fig. 4). Subsequently, based on the sum of SUCRA of OS and relapse, systematic cluster analysis divides the CCRT into a cluster, further supporting this strategy as the best option (Fig. 5).

Further OLAP cube analysis demonstrated that when using a three-category approach, CCRT and RS were divided into a single group, indicating CCRT to be the optimal intervention and RS to be the worst (Table 3, Fig. 5).

**Table 3:**
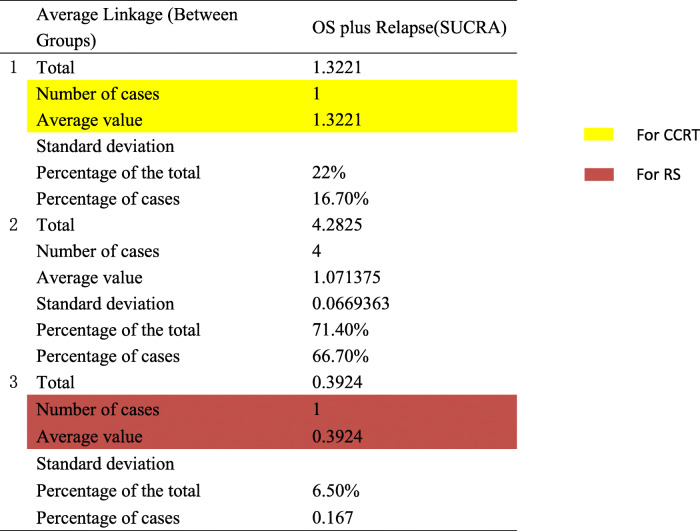
OLAP cube analysis of OS plus relapse

*OS* Overall survival, *SUCRA* Surface under the cumulative ranking curve

## Discussion

We performed an NMA study of treatments related to locally advanced cervical cancer in women to assess the relative effectiveness of various treatments in trials to date. Among all interventions evaluated, CCRT demonstrated the highest comprehensive efficacy, as evidenced by the sum of SUCRA value. After Bayesian analysis, a systematic cluster analysis was performed to determine the treatment interventions that can be evenly grouped according to the sum of SUCRA values ​​of the two endpoints obtained by NMA, setting the cluster numbers to 2–5 categories to facilitate observation. At 3 clusters, CCRT and RS are classified into different groups. From the SUCRA value, it is apparent that the top-ranked treatments vary depending on the endpoint of the assessment. The sum of the SUCRA value of each of the two endpoints implies that CCRT is the optimal intervention for FIGO stage IB2/IIA2 cervical tumor. Hierarchical cluster analysis further verified that the CCRT separated into an independent group. Therefore, in FIGO stage IB2/IIA2 cervical cancer, CCRT appears the optimal management strategy for cases.

Cervical cancer is a serious women’s health issue worldwide; most cervical tumors are caused by high-risk human papillomavirus (HR-HPV) infection [41]. An appreciable proportion of cervical cancer is diagnosed at FIGO stage IB2/IIA2. Previous reports have compared these cases against stage IB1 disease, reporting an increased risk of death from FIGO stage IB2 cervical cancer disease representing a close-to-doubling of risk (HR 1.98, 95% CI 1.62–2.41, *P* < 0.001) [42]. Optimal management of these cases is therefore crucial.

The efficacy of CCRT in the treatment of locally advanced cervical cancer has been compared in previous randomized controlled trials or meta-analysis; these studies have suggested the superiority of CCRT versus other regimens [43–45]. Gupta et al. [31] suggested that in locally advanced cervical cancer, cisplatin-based concurrent radiotherapy and chemotherapy can achieve better disease-free survival compared with radical surgery after neoadjuvant chemotherapy.

Other studies suggest that - although only significant for patients with stage IB2-IIB - NACT plus RS seems to confer survival benefit compared to RT [34]. Compared with RS alone, especially compared with CCRT, NACT + RS may improve the long-term disease-free survival rate and overall survival rate of patients with locally advanced cervical cancer stage IB2-IIB [7]. Moreover, total hysterectomy after NACT may be an option for patients with stage Ib2-IIb cervical adenocarcinoma [46]. However, this study found that NACT did not improve overall survival, but reduced the number of patients receiving postoperative radiotherapy [47]. Lee et al. [48] described no therapeutic advantage of NACT + RS compared to CCRT. Some scholars believe that preoperative brachytherapy in the vaginal cavity can be used as an effective treatment method for comprehensive treatment of stage Ib2 and IIa cervical cancer, with a satisfactory local control rate for stage Ib2 and IIa cervical cancer [32]. The findings of Landoni et al. [13] indicate that, in terms of survival, there is no alternative treatment for early cervical cancer. Long-term follow-up confirmed that the best treatment for individual patients should take into account clinical factors, such as menopausal status, comorbidities, histological type, and tumor diameter. In light of our findings in the context of this controversy, CCRT appears to be the most appropriate therapeutic option.

NMA comes with conceptual and technical considerations [49], including the need to meet transitivity and consistency assumptions. The transitivity hypothesis means that the diverse treatments in all studies are comparable in terms of the characteristics that may affect the results. In order to ensure transmissibility, except for treatment interventions, other aspects of the included study should be relatively similar [49, 50]. In order to meet this transitivity assumption, we limited the study to locally advanced cervical cancer.

Consistency described the statistical consistency between the direct comparison and the indirect comparison of each paired comparison in NMA. Differences indicate inconsistency [19, 29, 49]. We use the confidence interval in the network Meta-analysis to test the heterogeneity and consistency of the two endpoints and use the node splitting method to detect local inconsistencies [30]. No major heterogeneity or consistency issues were identified in the OS or relapse analysis.

The advantage of this study is that our NMA compares each intervention for locally advanced cervical cancer. At present, the treatment of stage IB2/IIA2 cervical cancer is still controversial; our findings are therefore of clear clinical interest.

We acknowledge several limitations of our study. We acknowledge the subjectivity of the risk bias assessment. Some of the include studies lacked blinding of participating subjects, personnel or external reviewers. Moreover, some studies had incomplete outcome data. One randomized control trial demonstrated higher risk, which originated from allocation concealment and double-blind design. The quality of several studies may have affected our analysis. In addition, due to incomplete data, very few data were available, so the endpoint of complication rate and type of different treatments were lacking. Another limitation of the study is that all 13 studies included cervical cancer stage IB2/IIA2, but a few studies not only included cervical cancer stage IB2 /IIA2. This may have some impact on our research.

## Conclusions

We report an analysis of all RCTs using different interventions in FIGO IB2/IIA2 cervical cancer; NMA identified that, in terms of effectiveness and safety, overall survival and relapse, CCRT may be the optimal treatment strategy in locally advanced cervical cancer. RS alone may be the least effective strategy. However, since these interventions have not yet been directly compared face-to-face, additional verification is necessary for the Phase 3 multicenter randomized controlled trial.

## Supplementary Information

**Additional file 1.**

**Additional file 2.**

**Additional file 3.**

**Additional file 4.**

**Additional file 5.**

**Additional file 6.**

**Additional file 7.**

**Additional file 8.**

**Additional file 9.**

## Data Availability

All data generated or analyzed during this study are included in this published article.
