# Supplementary material for: Effectiveness comparisons of various therapies for FIGO stage IB2/IIA2 cervical cancer: a Bayesian network meta-analysis
Source: BMC Cancer. 2021 Oct 6;21:1078. doi: 10.1186/s12885-021-08685-9 (PMC8493709; doi:10.1186/s12885-021-08685-9)
Supplement: Supplementary file 1 — Additional file 1. [file 12885_2021_8685_MOESM1_ESM.pdf]

The Specific Search Strategy

Pubmed search strategy:

#1 "Uterine Cervical Neoplasms"[Mesh]
#2 (((((((((((((((((((((((((Cervical Neoplasm, Uterine[Title/Abstract]) OR (Cervical Neoplasms, Uterine[Title/Abstract])) OR (Neoplasm, Uterine Cervical[Title/Abstract])) OR (Neoplasms, Uterine Cervical[Title/Abstract])) OR (Uterine Cervical Neoplasm[Title/Abstract])) OR (Neoplasms, Cervical[Title/Abstract])) OR (Cervical Neoplasms[Title/Abstract])) OR (Cervical Neoplasm[Title/Abstract])) OR (Neoplasm, Cervical[Title/Abstract])) OR (Neoplasms, Cervix[Title/Abstract])) OR (Cervix Neoplasms[Title/Abstract])) OR (Cervix Neoplasm[Title/Abstract])) OR (Neoplasm, Cervix[Title/Abstract])) OR (Cancer of the Uterine Cervix[Title/Abstract])) OR (Cancer of the Cervix[Title/Abstract])) OR (Cervical Cancer[Title/Abstract])) OR (Uterine Cervical Cancer[Title/Abstract])) OR (Cancer, Uterine Cervical[Title/Abstract])) OR (Cancers, Uterine Cervical[Title/Abstract])) OR (Cervical Cancer, Uterine[Title/Abstract])) OR (Cervical Cancers, Uterine[Title/Abstract])) OR (Uterine Cervical Cancers[Title/Abstract])) OR (Cancer of Cervix[Title/Abstract])) OR (Cervix Cancer[Title/Abstract])) OR (Cancer, Cervix[Title/Abstract])) OR (Cancers, Cervix[Title/Abstract])
#3 ("Uterine Cervical Neoplasms"[Mesh]) OR ((((((((((((((((((((((((((Cervical Neoplasm, Uterine[Title/Abstract]) OR (Cervical Neoplasms, Uterine[Title/Abstract])) OR (Neoplasm, Uterine Cervical[Title/Abstract])) OR (Neoplasms, Uterine Cervical[Title/Abstract])) OR (Uterine Cervical Neoplasm[Title/Abstract])) OR (Neoplasms, Cervical[Title/Abstract])) OR (Cervical Neoplasms[Title/Abstract])) OR (Cervical Neoplasm[Title/Abstract])) OR (Neoplasm, Cervical[Title/Abstract])) OR (Neoplasms, Cervix[Title/Abstract])) OR (Cervix Neoplasms[Title/Abstract])) OR (Cervix Neoplasm[Title/Abstract])) OR (Neoplasm, Cervix[Title/Abstract])) OR (Cancer of the Uterine Cervix[Title/Abstract])) OR (Cancer of the Cervix[Title/Abstract])) OR (Cervical Cancer[Title/Abstract])) OR (Uterine Cervical Cancer[Title/Abstract])) OR (Cancer, Uterine Cervical[Title/Abstract])) OR (Cancers, Uterine Cervical[Title/Abstract])) OR (Cervical Cancer, Uterine[Title/Abstract])) OR (Cervical Cancers, Uterine[Title/Abstract])) OR (Uterine Cervical Cancers[Title/Abstract])) OR (Cancer of Cervix[Title/Abstract])) OR (Cervix Cancer[Title/Abstract])) OR (Cancer, Cervix[Title/Abstract])) OR (Cancers, Cervix[Title/Abstract]))
#4 "Chemoradiotherapy"[Mesh]
#5 ((((((((((((((((((((((Chemoradiotherapies[Title/Abstract]) OR (Radiochemotherapy[Title/Abstract])) OR (Radiochemotherapies[Title/Abstract])) OR (Concurrent Chemoradiotherapy[Title/Abstract])) OR (Chemoradiotherapies, Concurrent[Title/Abstract])) OR (Chemoradiotherapy, Concurrent[Title/Abstract])) OR (Concurrent Chemoradiotherapies[Title/Abstract])) OR (Synchronous Chemoradiotherapy[Title/Abstract])) OR (Chemoradiotherapies, Synchronous[Title/Abstract])) OR (Chemoradiotherapy, Synchronous[Title/Abstract])) OR (Synchronous Chemoradiotherapies[Title/Abstract])) OR (Concurrent

Radiochemotherapy[Title/Abstract])) OR (Concurrent Radiochemotherapies[Title/Abstract])) OR (Radiochemotherapies, Concurrent[Title/Abstract])) OR (Radiochemotherapy, Concurrent[Title/Abstract])) OR (Concomitant Chemoradiotherapy[Title/Abstract])) OR (Chemoradiotherapies, Concomitant[Title/Abstract])) OR (Chemoradiotherapy, Concomitant[Title/Abstract])) OR (Concomitant Chemoradiotherapies[Title/Abstract])) OR (Concomitant Radiochemotherapy[Title/Abstract])) OR (Concomitant Radiochemotherapies[Title/Abstract])) OR (Radiochemotherapies, Concomitant[Title/Abstract])) OR (Radiochemotherapy, Concomitant[Title/Abstract])

#6 CCRT[Title/Abstract]

#7 "General Surgery"[Mesh]

#8 (Surgery,General[Title/Abstract]) OR (Surgery[Title/Abstract])

#9 "Surgical Procedures, Operative"[Mesh]

#10 (((((((((Operative Surgical Procedure[Title/Abstract]) OR (Surgical Procedure, Operative[Title/Abstract])) OR (Procedure, Operative Surgical[Title/Abstract])) OR (Procedures, Operative Surgical[Title/Abstract])) OR (Operative Procedures[Title/Abstract])) OR (Operative Procedure[Title/Abstract])) OR (Procedure, Operative[Title/Abstract])) OR (Procedures, Operative[Title/Abstract])) OR (Operative Surgical Procedures[Title/Abstract])) OR (Surgery, Ghost[Title/Abstract])) OR (Ghost Surgery[Title/Abstract])

#11 "Gynecologic Surgical Procedures"[Mesh]

#12 ((((((((((((((((Procedures, Gynecologic Surgical[Title/Abstract]) OR (Surgical Procedure, Gynecologic[Title/Abstract])) OR (Surgery, Gynecological[Title/Abstract])) OR (Gynecological Surgeries[Title/Abstract])) OR (Gynecological Surgery[Title/Abstract])) OR (Surgeries, Gynecological[Title/Abstract])) OR (Gynecologic Surgical Procedure[Title/Abstract])) OR (Surgical Procedures, Gynecologic[Title/Abstract])) OR (Gynecological Surgical Procedure[Title/Abstract])) OR (Gynecological Surgical Procedures[Title/Abstract])) OR (Procedure, Gynecological Surgical[Title/Abstract])) OR (Procedures, Gynecological Surgical[Title/Abstract])) OR (Surgical Procedure, Gynecological[Title/Abstract])) OR (Surgical Procedures, Gynecological[Title/Abstract])) OR (Procedure, Gynecologic Surgical[Title/Abstract])) OR (Gynecologic Surgery[Title/Abstract])) OR (Gynecologic Surgeries[Title/Abstract])) OR (Surgeries, Gynecologic[Title/Abstract])) OR (Surgery, Gynecologic[Title/Abstract])

#13 "Hysterectomy"[Mesh]

#14 Hysterectomies[Title/Abstract]

#15 "Chemotherapy, Adjuvant"[Mesh]

#16 ((Adjuvant Chemotherapy[Title/Abstract]) OR (Drug Therapy,Adjuvant [Title/Abstract])) OR (Adjuvant Drug Therapy[Title/Abstract])

#17 neoadjuvant chemotherapy[Title/Abstract]

#18 "Drug Therapy"[Mesh]

#19 ((((((Therapy, Drug[Title/Abstract]) OR (Drug Therapies[Title/Abstract])) OR (Therapies, Drug[Title/Abstract])) OR (Chemotherapy[Title/Abstract])) OR (Chemotherapies[Title/Abstract])) OR (Pharmacotherapy[Title/Abstract])) OR (Pharmacotherapies[Title/Abstract])

#20 "Radiotherapy"[Mesh]
#21 ((((((((((((((((Radiotherapies[Title/Abstract]) OR (Radiation Therapy[Title/Abstract])) OR (Radiation Therapies[Title/Abstract])) OR (Therapies, Radiation[Title/Abstract])) OR (Therapy, Radiation[Title/Abstract])) OR (Radiation Treatment[Title/Abstract])) OR (Radiation Treatments[Title/Abstract])) OR (Treatment, Radiation[Title/Abstract])) OR (Radiotherapy, Targeted[Title/Abstract])) OR (Radiotherapies, Targeted[Title/Abstract])) OR (Targeted Radiotherapies[Title/Abstract])) OR (Targeted Radiotherapy[Title/Abstract])) OR (Targeted Radiation Therapy[Title/Abstract])) OR (Radiation Therapies, Targeted[Title/Abstract])) OR (Targeted Radiation Therapies[Title/Abstract])) OR (Therapies, Targeted Radiation[Title/Abstract])) OR (Therapy, Targeted Radiation[Title/Abstract])) OR (Radiation Therapy, Targeted[Title/Abstract])
#22 ((preoperative radiation[Title/Abstract]) OR (preoperative treatment[Title/Abstract])) OR (preoperative radiotherapy[Title/Abstract])
#23 (((((((((((((((("Chemoradiotherapy"[Mesh] OR ((((((((((((((((((((((Chemoradiotherapies[Title/Abstract]) OR (Radiochemotherapy[Title/Abstract])) OR (Radiochemotherapies[Title/Abstract])) OR (Concurrent Chemoradiotherapy[Title/Abstract])) OR (Chemoradiotherapies, Concurrent[Title/Abstract])) OR (Chemoradiotherapy, Concurrent[Title/Abstract])) OR (Concurrent Chemoradiotherapies[Title/Abstract])) OR (Synchronous Chemoradiotherapy[Title/Abstract])) OR (Chemoradiotherapies, Synchronous[Title/Abstract])) OR (Chemoradiotherapy, Synchronous[Title/Abstract])) OR (Synchronous Chemoradiotherapies[Title/Abstract])) OR (Concurrent Radiochemotherapy[Title/Abstract])) OR (Concurrent Radiochemotherapies[Title/Abstract])) OR (Radiochemotherapies, Concurrent[Title/Abstract])) OR (Radiochemotherapy, Concurrent[Title/Abstract])) OR (Concomitant Chemoradiotherapy[Title/Abstract])) OR (Chemoradiotherapies, Concomitant[Title/Abstract])) OR (Chemoradiotherapy, Concomitant[Title/Abstract])) OR (Concomitant Chemoradiotherapies[Title/Abstract])) OR (Concomitant Radiochemotherapy[Title/Abstract])) OR (Concomitant Radiochemotherapies[Title/Abstract])) OR (Radiochemotherapies, Concomitant[Title/Abstract])) OR (Radiochemotherapy, Concomitant[Title/Abstract]))) OR (CCRT[Title/Abstract])) OR ("General Surgery"[Mesh])) OR ((Surgery,General[Title/Abstract]) OR (Surgery[Title/Abstract]))) OR ((Surgery,General[Title/Abstract]) OR (Surgery[Title/Abstract]))) OR ("Surgical Procedures, Operative"[Mesh])) OR ((((((((((Operative Surgical Procedure[Title/Abstract]) OR (Surgical Procedure, Operative[Title/Abstract])) OR (Procedure, Operative Surgical[Title/Abstract])) OR (Procedures, Operative Surgical[Title/Abstract])) OR (Operative Procedures[Title/Abstract])) OR (Operative Procedure[Title/Abstract])) OR (Procedure, Operative[Title/Abstract])) OR (Procedures, Operative[Title/Abstract])) OR (Operative Surgical Procedures[Title/Abstract])) OR (Surgery, Ghost[Title/Abstract])) OR (Ghost Surgery[Title/Abstract]))) OR ("Gynecologic Surgical Procedures"[Mesh])) OR ((((((((((((((((((Procedures, Gynecologic Surgical[Title/Abstract]) OR (Surgical

Procedure, Gynecologic[Title/Abstract])) OR (Surgery, Gynecological[Title/Abstract])) OR (Gynecological Surgeries[Title/Abstract])) OR (Gynecological Surgery[Title/Abstract])) OR (Surgeries, Gynecological[Title/Abstract])) OR (Gynecologic Surgical Procedure[Title/Abstract])) OR (Surgical Procedures, Gynecologic[Title/Abstract])) OR (Gynecological Surgical Procedure[Title/Abstract])) OR (Gynecological Surgical Procedures[Title/Abstract])) OR (Procedure, Gynecological Surgical[Title/Abstract])) OR (Procedures, Gynecological Surgical[Title/Abstract])) OR (Surgical Procedure, Gynecological[Title/Abstract])) OR (Surgical Procedures, Gynecological[Title/Abstract])) OR (Procedure, Gynecologic Surgical[Title/Abstract])) OR (Gynecologic Surgery[Title/Abstract])) OR (Gynecologic Surgeries[Title/Abstract])) OR (Surgeries, Gynecologic[Title/Abstract])) OR (Surgery, Gynecologic[Title/Abstract])))) OR ("Hysterectomy"[Mesh])) OR (Hysterectomies[Title/Abstract])) OR ("Chemotherapy, Adjuvant"[Mesh])) OR (((Adjuvant Chemotherapy[Title/Abstract]) OR (Drug Therapy, Adjuvant[Title/Abstract])) OR (Adjuvant Drug Therapy[Title/Abstract]))) OR (neoadjuvant chemotherapy[Title/Abstract])) OR ("Drug Therapy"[Mesh])) OR (((((((Therapy, Drug[Title/Abstract]) OR (Drug Therapies[Title/Abstract])) OR (Therapies, Drug[Title/Abstract])) OR (Chemotherapy[Title/Abstract])) OR (Chemotherapies[Title/Abstract])) OR (Pharmacotherapy[Title/Abstract])) OR (Pharmacotherapies[Title/Abstract]))) OR ("Radiotherapy"[Mesh])) OR ((((((((((((((((((Radiotherapies[Title/Abstract]) OR (Radiation Therapy[Title/Abstract])) OR (Radiation Therapies[Title/Abstract])) OR (Therapies, Radiation[Title/Abstract])) OR (Therapy, Radiation[Title/Abstract])) OR (Radiation Treatment[Title/Abstract])) OR (Radiation Treatments[Title/Abstract])) OR (Treatment, Radiation[Title/Abstract])) OR (Radiotherapy, Targeted[Title/Abstract])) OR (Radiotherapies, Targeted[Title/Abstract])) OR (Targeted Radiotherapies[Title/Abstract])) OR (Targeted Radiotherapy[Title/Abstract])) OR (Targeted Radiation Therapy[Title/Abstract])) OR (Radiation Therapies, Targeted[Title/Abstract])) OR (Targeted Radiation Therapies[Title/Abstract])) OR (Therapies, Targeted Radiation[Title/Abstract])) OR (Therapy, Targeted Radiation[Title/Abstract])) OR (Radiation Therapy, Targeted[Title/Abstract]))) OR (((preoperative radiation[Title/Abstract]) OR (preoperative treatment[Title/Abstract])) OR (preoperative radiotherapy[Title/Abstract]))

#24 (Randomized Controlled Trial [Title/Abstract]) OR (Randomized [Title/Abstract]) OR (Randomized Controlled Trials [Title/Abstract]) OR (Randomised Controlled Trials [Title/Abstract]) OR (RCT [Title/Abstract]) OR (RCTs [Title/Abstract])

#25 ((("Uterine Cervical Neoplasms"[Mesh]) OR ((((((((((((((((((((((((((Cervical Neoplasm, Uterine[Title/Abstract]) OR (Cervical Neoplasms, Uterine[Title/Abstract])) OR (Neoplasm, Uterine Cervical[Title/Abstract])) OR (Neoplasms, Uterine Cervical[Title/Abstract])) OR (Uterine Cervical Neoplasm[Title/Abstract])) OR (Neoplasms, Cervical[Title/Abstract])) OR (Cervical Neoplasms[Title/Abstract])) OR (Cervical Neoplasm[Title/Abstract])) OR (Neoplasm, Cervical[Title/Abstract])) OR (Neoplasms, Cervix[Title/Abstract])) OR (Cervix Neoplasms[Title/Abstract])) OR (Cervix Neoplasm[Title/Abstract])) OR (Neoplasm, Cervix[Title/Abstract])) OR (Cancer

of the Uterine Cervix[Title/Abstract])) OR (Cancer of the Cervix[Title/Abstract])) OR (Cervical Cancer[Title/Abstract])) OR (Uterine Cervical Cancer[Title/Abstract])) OR (Cancer, Uterine Cervical[Title/Abstract])) OR (Cancers, Uterine Cervical[Title/Abstract])) OR (Cervical Cancer, Uterine[Title/Abstract])) OR (Cervical Cancers, Uterine[Title/Abstract])) OR (Uterine Cervical Cancers[Title/Abstract])) OR (Cancer of Cervix[Title/Abstract])) OR (Cervix Cancer[Title/Abstract])) OR (Cancer, Cervix[Title/Abstract])) OR (Cancers, Cervix[Title/Abstract]))) AND (((((((((((((((((((("Chemoradiotherapy"[Mesh]) OR (((((((((((((((((((((((Chemoradiotherapies[Title/Abstract]) OR (Radiochemotherapy[Title/Abstract])) OR (Radiochemotherapies[Title/Abstract])) OR (Concurrent Chemoradiotherapy[Title/Abstract])) OR (Chemoradiotherapies, Concurrent[Title/Abstract])) OR (Chemoradiotherapy, Concurrent[Title/Abstract])) OR (Concurrent Chemoradiotherapies[Title/Abstract])) OR (Synchronous Chemoradiotherapy[Title/Abstract])) OR (Chemoradiotherapies, Synchronous[Title/Abstract])) OR (Chemoradiotherapy, Synchronous[Title/Abstract])) OR (Synchronous Chemoradiotherapies[Title/Abstract])) OR (Concurrent Radiochemotherapy[Title/Abstract])) OR (Concurrent Radiochemotherapies[Title/Abstract])) OR (Radiochemotherapies, Concurrent[Title/Abstract])) OR (Radiochemotherapy, Concurrent[Title/Abstract])) OR (Concomitant Chemoradiotherapy[Title/Abstract])) OR (Chemoradiotherapies, Concomitant[Title/Abstract])) OR (Chemoradiotherapy, Concomitant[Title/Abstract])) OR (Concomitant Chemoradiotherapies[Title/Abstract])) OR (Concomitant Radiochemotherapy[Title/Abstract])) OR (Concomitant Radiochemotherapies[Title/Abstract])) OR (Radiochemotherapies, Concomitant[Title/Abstract])) OR (Radiochemotherapy, Concomitant[Title/Abstract])))) OR (CCRT[Title/Abstract])) OR ("General Surgery"[Mesh])) OR ((Surgery,General[Title/Abstract]) OR (Surgery[Title/Abstract]))) OR ((Surgery,General[Title/Abstract]) OR (Surgery[Title/Abstract]))) OR ("Surgical Procedures, Operative"[Mesh])) OR (((((((((((Operative Surgical Procedure[Title/Abstract]) OR (Surgical Procedure, Operative[Title/Abstract])) OR (Procedure, Operative Surgical[Title/Abstract])) OR (Procedures, Operative Surgical[Title/Abstract])) OR (Operative Procedures[Title/Abstract])) OR (Operative Procedure[Title/Abstract])) OR (Procedure, Operative[Title/Abstract])) OR (Procedures, Operative[Title/Abstract])) OR (Operative Surgical Procedures[Title/Abstract])) OR (Surgery, Ghost[Title/Abstract])) OR (Ghost Surgery[Title/Abstract]))) OR ("Gynecologic Surgical Procedures"[Mesh])) OR (((((((((((((((((((Procedures, Gynecologic Surgical[Title/Abstract]) OR (Surgical Procedure, Gynecologic[Title/Abstract])) OR (Surgery, Gynecological[Title/Abstract])) OR (Gynecological Surgeries[Title/Abstract])) OR (Gynecological Surgery[Title/Abstract])) OR (Surgeries, Gynecological[Title/Abstract])) OR (Gynecologic Surgical Procedure[Title/Abstract])) OR (Surgical Procedures, Gynecologic[Title/Abstract])) OR (Gynecological Surgical Procedure[Title/Abstract])) OR (Gynecological Surgical Procedures[Title/Abstract])) OR (Procedure, Gynecological Surgical[Title/Abstract])) OR (Procedures, Gynecological Surgical[Title/Abstract])) OR

(Surgical Procedure, Gynecological[Title/Abstract])) OR (Surgical Procedures, Gynecological[Title/Abstract])) OR (Procedure, Gynecologic Surgical[Title/Abstract])) OR (Gynecologic Surgery[Title/Abstract])) OR (Gynecologic Surgeries[Title/Abstract])) OR (Surgeries, Gynecologic[Title/Abstract])) OR (Surgery, Gynecologic[Title/Abstract]))) OR ("Hysterectomy"[Mesh])) OR (Hysterectomies[Title/Abstract])) OR ("Chemotherapy, Adjuvant"[Mesh])) OR (((Adjuvant Chemotherapy[Title/Abstract]) OR (Drug Therapy, Adjuvant[Title/Abstract])) OR (Adjuvant Drug Therapy[Title/Abstract]))) OR (neoadjuvant chemotherapy[Title/Abstract])) OR ("Drug Therapy"[Mesh])) OR ((((((Therapy, Drug[Title/Abstract]) OR (Drug Therapies[Title/Abstract])) OR (Therapies, Drug[Title/Abstract])) OR (Chemotherapy[Title/Abstract])) OR (Chemotherapies[Title/Abstract])) OR (Pharmacotherapy[Title/Abstract])) OR (Pharmacotherapies[Title/Abstract]))) OR ("Radiotherapy"[Mesh])) OR ((((((((((((((((Radiotherapies[Title/Abstract]) OR (Radiation Therapy[Title/Abstract])) OR (Radiation Therapies[Title/Abstract])) OR (Therapies, Radiation[Title/Abstract])) OR (Therapy, Radiation[Title/Abstract])) OR (Radiation Treatment[Title/Abstract])) OR (Radiation Treatments[Title/Abstract])) OR (Treatment, Radiation[Title/Abstract])) OR (Radiotherapy, Targeted[Title/Abstract])) OR (Radiotherapies, Targeted[Title/Abstract])) OR (Targeted Radiotherapies[Title/Abstract])) OR (Targeted Radiotherapy[Title/Abstract])) OR (Targeted Radiation Therapy[Title/Abstract])) OR (Radiation Therapies, Targeted[Title/Abstract])) OR (Targeted Radiation Therapies[Title/Abstract])) OR (Therapies, Targeted Radiation[Title/Abstract])) OR (Therapy, Targeted Radiation[Title/Abstract])) OR (Radiation Therapy, Targeted[Title/Abstract]))) OR (((preoperative radiation[Title/Abstract]) OR (preoperative treatment[Title/Abstract])) OR (preoperative radiotherapy[Title/Abstract])))) AND ((Randomized Controlled Trial [Title/Abstract]) OR (Randomized [Title/Abstract]) OR (Randomized Controlled Trials [Title/Abstract]) OR (Randomised Controlled Trials [Title/Abstract]) OR (RCT [Title/Abstract]) OR (RCTs [Title/Abstract]))

Embase search strategy:

#1 'uterine cervix tumor'/exp
#2 'cervical neoplasm, uterine':ti,ab
#3 'cervical neoplasms, uterine':ti,ab
#4 'neoplasm, uterine cervical':ti,ab
#5 'neoplasms, uterine cervical':ti,ab
#6 'uterine cervical neoplasm':ti,ab
#7 'neoplasms, cervical':ti,ab
#8 'cervical neoplasms':ti,ab
#9 'cervical neoplasm':ti,ab
#10 'neoplasm, cervical':ti,ab

#11 'neoplasms, cervix':ti,ab
#12 'cervix neoplasms':ti,ab
#13 'cervix neoplasm':ti,ab
#14 'neoplasm, cervix':ti,ab
#15 'cancer of the uterine cervix':ti,ab
#16 'cancer of the cervix':ti,ab
#17 'cervical cancer':ti,ab
#18 'uterine cervical cancer':ti,ab
#19 'cancer, uterine cervical':ti,ab
#20 'cancers, uterine cervical':ti,ab
#21 'cervical cancer, uterine':ti,ab
#22 'cervical cancers, uterine':ti,ab
#23 'uterine cervical cancers':ti,ab
#24 'cancer of cervix':ti,ab
#25 'cervix cancer':ti,ab
#26 'cancer, cervix':ti,ab
#27 'cancers, cervix':ti,ab
#28 #1 OR #2 OR #3 OR #4 OR #5 OR #6 OR #7 OR #8 OR #9 OR #10 OR #11 OR #12 OR #13 OR #14 OR #15 OR #16 OR #17 OR #18 OR #19 OR #20 OR #21 OR #22 OR #23 OR #24 OR #25 OR #26 OR #27
#29 'chemoradiotherapy'/exp
#30 'chemoradiotherapies':ti,ab
#31 'radiochemotherapy':ti,ab
#32 'radiochemotherapies':ti,ab
#33 'concurrent chemoradiotherapy':ti,ab
#34 'chemoradiotherapies, concurrent':ti,ab
#35 'chemoradiotherapy, concurrent':ti,ab
#36 'concurrent chemoradiotherapies':ti,ab
#37 'synchronous chemoradiotherapy':ti,ab
#38 'chemoradiotherapies, synchronous':ti,ab
#39 'chemoradiotherapy, synchronous':ti,ab
#40 'synchronous chemoradiotherapies':ti,ab
#41 'concurrent radiochemotherapy':ti,ab
#42 'concurrent radiochemotherapies':ti,ab
#43 'radiochemotherapies, concurrent':ti,ab
#44 'radiochemotherapy, concurrent':ti,ab
#45 'concomitant chemoradiotherapy':ti,ab
#46 'chemoradiotherapies, concomitant':ti,ab
#47 'chemoradiotherapy, concomitant':ti,ab
#48 'concomitant chemoradiotherapies':ti,ab
#49 'concomitant radiochemotherapy':ti,ab
#50 'concomitant radiochemotherapies':ti,ab
#51 'radiochemotherapies, concomitant':ti,ab
#52 'radiochemotherapy, concomitant':ti,ab

#53 'ccrt':ti,ab
#54 'general surgery'/exp
#55 'surgery,general':ti,ab
#56 'surgery':ti,ab
#57 'surgery'/exp
#58 'operative surgical procedure':ti,ab
#59 'surgical procedure, operative':ti,ab
#60 'procedure, operative surgical':ti,ab
#61 'procedures, operative surgical':ti,ab
#62 'operative procedures':ti,ab
#63 'operative procedure':ti,ab
#64 'procedure, operative':ti,ab
#65 'procedures, operative':ti,ab
#66 'operative surgical procedures':ti,ab
#67 'surgery, ghost':ti,ab
#68 'ghost surgery':ti,ab
#69 'gynecologic surgery'/exp
#70 'procedures, gynecologic surgical':ti,ab
#71 'surgical procedure, gynecologic':ti,ab
#72 'surgery, gynecological':ti,ab
#73 'gynecological surgeries':ti,ab
#74 'gynecological surgery':ti,ab
#75 'surgeries, gynecological':ti,ab
#76 'gynecologic surgical procedure':ti,ab
#77 'surgical procedures, gynecologic':ti,ab
#78 'gynecological surgical procedure':ti,ab
#79 'gynecological surgical procedures':ti,ab
#80 'procedure, gynecological surgical':ti,ab
#81 'procedures, gynecological surgical':ti,ab
#82 'surgical procedure, gynecological':ti,ab
#83 'surgical procedures, gynecological':ti,ab
#84 'procedure, gynecologic surgical':ti,ab
#85 'gynecologic surgery':ti,ab
#86 'gynecologic surgeries':ti,ab
#87 'surgeries, gynecologic':ti,ab
#88 'surgery, gynecologic':ti,ab
#89 'hysterectomy'/exp
#90 'hysterectomies':ti,ab
#91 'adjuvant chemotherapy'/exp
#92 'neoadjuvant chemotherapy'/exp
#93 'adjuvant chemotherapy':ti,ab
#94 'drug therapy, adjuvant':ti,ab
#95 'adjuvant drug therapy':ti,ab
#96 'neoadjuvant chemotherapy':ti,ab

#97 'drug therapy'/exp
#98 'therapy, drug':ti,ab
#99 'drug therapies':ti,ab
#100 'therapies, drug':ti,ab
#101 'chemotherapy':ti,ab
#102 'chemotherapies':ti,ab
#103 'pharmacotherapy':ti,ab
#104 'pharmacotherapies':ti,ab
#105 'radiotherapy'/exp
#106 'radiotherapies':ti,ab
#107 'radiation therapy':ti,ab
#108 'radiation therapies':ti,ab
#109 'therapies, radiation':ti,ab
#110 'therapy, radiation':ti,ab
#111 'radiation treatment':ti,ab
#112 'radiation treatments':ti,ab
#113 'treatment, radiation':ti,ab
#114 'radiotherapy, targeted':ti,ab
#115 'radiotherapies, targeted':ti,ab
#116 'targeted radiotherapies':ti,ab
#117 'targeted radiotherapy':ti,ab
#118 'targeted radiation therapy':ti,ab
#119 'radiation therapies, targeted':ti,ab
#120 'targeted radiation therapies':ti,ab
#121 'therapies, targeted radiation':ti,ab
#122 'therapy, targeted radiation':ti,ab
#123 'radiation therapy, targeted':ti,ab
#124 'preoperative radiotherapy':ti,ab
#125 'preoperative treatment':ti,ab
#126 'preoperative radiotherapy':ti,ab
#127 #29 OR #30 OR #31 OR #32 OR #33 OR #34 OR #35 OR #36 OR #37 OR #38 OR #39 OR #40 OR #41 OR #42 OR #43 OR #44 OR #45 OR #46 OR #47 OR #48 OR #49 OR #50 OR #51 OR #52 OR #53 OR #54 OR #55 OR #56 OR #57 OR #58 OR #59 OR #60 OR #61 OR #62 OR #63 OR #64 OR #65 OR #66 OR #67 OR #68 OR #69 OR #70 OR #71 OR #72 OR #73 OR #74 OR #75 OR #76 OR #77 OR #78 OR #79 OR #80 OR #81 OR #82 OR #83 OR #84 OR #85 OR #86 OR #87 OR #88 OR #89 OR #90 OR #91 OR #92 OR #93 OR #94 OR #95 OR #96 OR #97 OR #98 OR #99 OR #100 OR #101 OR #102 OR #103 OR #104 OR #105 OR #106 OR #107 OR #108 OR #109 OR #110 OR #111 OR #112 OR #113 OR #114 OR #115 OR #116 OR #117 OR #118 OR #119 OR #120 OR #121 OR #122 OR #123 OR #124 OR #125 OR #126
#128 'randomized controlled trial':ti,ab
#129 'randomized':ti,ab
#130 'randomized controlled trials':ti,ab
#131 'randomised controlled trials':ti,ab

#132 'rct':ti,ab
#133 'rcts':ti,ab
#134 #128 OR #129 OR #130 OR #131 OR #132 OR #133
#135 #28 AND #127 AND #134

cochrane search strategy:

#1 MeSH descriptor: [Uterine Cervical Neoplasms] explode all trees
#2 Cervical Neoplasm, Uterine:ti,ab,kw OR Cervical Neoplasms, Uterine:ti,ab,kw OR Neoplasm, Uterine Cervical:ti,ab,kw OR Neoplasms, Uterine Cervical:ti,ab,kw OR Uterine Cervical Neoplasm:ti,ab,kw OR Neoplasms, Cervical:ti,ab,kw OR Cervical Neoplasms:ti,ab,kw OR Cervical Neoplasm:ti,ab,kw OR Neoplasm, Cervical:ti,ab,kw OR Neoplasms, Cervix:ti,ab,kw OR Cervix Neoplasms:ti,ab,kw OR Cervix Neoplasm:ti,ab,kw OR Neoplasm, Cervix:ti,ab,kw OR Cancer of the Uterine Cervix:ti,ab,kw OR Cancer of the Cervix:ti,ab,kw OR Cervical Cancer:ti,ab,kw OR Uterine Cervical Cancer:ti,ab,kw OR Cancer, Uterine Cervical:ti,ab,kw OR Cancers, Uterine Cervical:ti,ab,kw OR Cervical Cancer, Uterine:ti,ab,kw OR Cervical Cancers, Uterine:ti,ab,kw OR Uterine Cervical Cancers:ti,ab,kw OR Cancer of Cervix:ti,ab,kw OR Cervix Cancer:ti,ab,kw OR Cancer, Cervix:ti,ab,kw OR Cancers, Cervix:ti,ab,kw
#3 #1 OR #2
#4 MeSH descriptor: [Chemoradiotherapy] explode all trees
#5 Chemoradiotherapies:ti,ab,kw OR Radiochemotherapy:ti,ab,kw OR Radiochemotherapies:ti,ab,kw OR Concurrent Chemoradiotherapy:ti,ab,kw OR Chemoradiotherapies, Concurrent:ti,ab,kw OR Chemoradiotherapy, Concurrent:ti,ab,kw OR Concurrent Chemoradiotherapies:ti,ab,kw OR Synchronous Chemoradiotherapy:ti,ab,kw OR Chemoradiotherapies, Synchronous:ti,ab,kw OR Chemoradiotherapy, Synchronous:ti,ab,kw OR Synchronous Chemoradiotherapies:ti,ab,kw OR Concurrent Radiochemotherapy:ti,ab,kw OR Concurrent Radiochemotherapies:ti,ab,kw OR Radiochemotherapies, Concurrent:ti,ab,kw OR Radiochemotherapy, Concurrent:ti,ab,kw OR Concomitant Chemoradiotherapy:ti,ab,kw OR Chemoradiotherapies, Concomitant:ti,ab,kw OR Chemoradiotherapy, Concomitant:ti,ab,kw OR Concomitant Chemoradiotherapies:ti,ab,kw OR Concomitant Radiochemotherapy:ti,ab,kw OR Concomitant Radiochemotherapies:ti,ab,kw OR Radiochemotherapies, Concomitant:ti,ab,kw OR
Radiochemotherapy, Concomitant:ti,ab,kw OR CCRT:ti,ab,kw
#6 MeSH descriptor: [General Surgery] explode all trees
#7 Surgery,General:ti,ab,kw OR Surgery:ti,ab,kw
#8 MeSH descriptor: [Surgical Procedures, Operative] explode all trees
#9 Operative Surgical Procedurel:ti,ab,kw OR Surgical Procedure, Operative:ti,ab,kw OR Procedure, Operative Surgical:ti,ab,kw OR Procedures, Operative Surgical:ti,ab,kw OR Operative Procedures:ti,ab,kw OR Operative Procedure:ti,ab,kw OR Procedure, Operative:ti,ab,kw OR Procedures,

Operative:ti,ab,kw OR Operative Surgical Procedures:ti,ab,kw OR Surgery, Ghost:ti,ab,kw OR Ghost Surgery:ti,ab,kw
#10 MeSH descriptor: [Gynecologic Surgical Procedures] explode all trees
#11 Procedures, Gynecologic Surgical:ti,ab,kw OR Surgical Procedure, Gynecologic:ti,ab,kw OR Surgery, Gynecological:ti,ab,kw OR Gynecological Surgeries:ti,ab,kw OR Gynecological Surgery:ti,ab,kw OR Surgeries, Gynecological:ti,ab,kw OR Gynecologic Surgical Procedure:ti,ab,kw OR Surgical Procedures, Gynecologic:ti,ab,kw OR Gynecological Surgical Procedure:ti,ab,kw OR Gynecological Surgical Procedures:ti,ab,kw OR Procedure, Gynecological Surgical:ti,ab,kw OR Procedures, Gynecological Surgical:ti,ab,kw OR Surgical Procedure, Gynecological:ti,ab,kw OR Surgical Procedures, Gynecological:ti,ab,kw OR Procedure, Gynecologic Surgical:ti,ab,kw OR Gynecologic Surgery:ti,ab,kw OR Gynecologic Surgeries:ti,ab,kw OR Surgeries, Gynecologic:ti,ab,kw OR Surgery, Gynecologic:ti,ab,kw
#12 MeSH descriptor: [Hysterectomy] explode all trees
#13 Hysterectomies:ti,ab,kw
#14 MeSH descriptor: [Chemotherapy, Adjuvant] explode all trees
#15 Adjuvant Chemotherapy:ti,ab,kw OR Drug Therapy, Adjuvant:ti,ab,kw OR Adjuvant Drug Therapy:ti,ab,kw OR neoadjuvant chemotherapy:ti,ab,kw
#16 MeSH descriptor: [Drug Therapy] explode all trees
#17 Therapy, Drug:ti,ab,kw OR Drug Therapies:ti,ab,kw OR Therapies, Drug:ti,ab,kw OR Chemotherapy:ti,ab,kw OR Chemotherapies:ti,ab,kw OR Pharmacotherapy:ti,ab,kw OR Pharmacotherapies:ti,ab,kw
#18 MeSH descriptor: [Radiotherapy] explode all trees
#19 Radiotherapies:ti,ab,kw OR Radiation Therapy:ti,ab,kw OR Radiation Therapies:ti,ab,kw OR Therapies, Radiation:ti,ab,kw OR Therapy, Radiation:ti,ab,kw OR Radiation Treatment:ti,ab,kw OR Radiation Treatments:ti,ab,kw OR Treatment, Radiation:ti,ab,kw OR Radiotherapy, Targeted:ti,ab,kw OR Radiotherapies, Targeted:ti,ab,kw OR Targeted Radiotherapies:ti,ab,kw OR Targeted Radiotherapy:ti,ab,kw OR Targeted Radiation Therapy:ti,ab,kw OR Radiation Therapies, Targeted:ti,ab,kw OR Targeted Radiation Therapies:ti,ab,kw OR Therapies, Targeted Radiation:ti,ab,kw OR Therapy, Targeted Radiation:ti,ab,kw OR Radiation Therapy, Targeted:ti,ab,kw OR preoperative radiation:ti,ab,kw OR preoperative treatment:ti,ab,kw OR preoperative radiotherapy:ti,ab,kw
#20 #4 OR #5 OR #6 OR #7 OR #8 OR #9 OR #10 OR #11 OR #12 OR #13 OR #14 OR #15 OR #16 OR #17 OR #18 OR #19
#21 #3 AND #20
