# Supplementary material for: Effectiveness comparisons of various therapies for FIGO stage IB2/IIA2 cervical cancer: a Bayesian network meta-analysis
Source: BMC Cancer. 2021 Oct 6;21:1078. doi: 10.1186/s12885-021-08685-9 (PMC8493709; doi:10.1186/s12885-021-08685-9)
Supplement: Supplementary file 2 — Additional file 2. [file 12885_2021_8685_MOESM2_ESM.pdf]

PRISMA NMA Checklist of Items to Include When Reporting A Systematic Review

Section/Topic	Item #	Checklist Item	Reported on Page #
**TITLE**			
Title	1	Identify the report as a systematic review incorporating a network meta-analysis (or related form ofmeta-analysis)	
**ABSTRACT**			
Structured summary	2	Provide a structured summary including, asapplicable: **Background**: main objectives **Methods**: data sources; study eligibility criteria, participants,and interventions; study appraisal; and synthesis methods,such as network meta-analysis. **Results**: number of studies and participants identified;summary estimates with corresponding confidence/credible intervals; treatment rankings may also be discussed.Authors may choose to summarize pairwise comparisons against a chosen treatment included in their analyses forbrevity. **Discussion/Conclusions**: limitations; conclusions and implications offindings. **Other**: primary source of funding; systematic review registration number with registry name.	Page. 1
**INTRODUCTION**			
Rationale	3	Describe the rationale for the review in the context of whatis already known, including mention of why a network meta-analysis has been conducted.	**Page.1-2**
Objectives	4	Provide an explicit statement of questions being addressed,with reference to participants, interventions, comparisons,outcomes, and study design(PICOS).	**Page.3**
**METHODS**			
Protocol and registration	5	Indicate whether a review protocol exists and if and where it can be accessed (e.g., Web address); and, if available, provide registration information,including registrationnumber.	**Page.2**
Eligibility criteria	6	Specify study characteristics (e.g., PICOS, length of follow-up)and report characteristics (e.g., years considered, language,publication status) used as criteria for eligibility, giving rationale. Clearly describe eligible treatments included in the treatment network, and note whether any have been clusteredor merged into the same node (withjustification).	**Page. 3 and Supplement Table.1**

Information sources	7	Describe all information sources (e.g., databases with datesof coverage, contact with study authors to identify additionalstudies) in the search and date last searched.	**Page.2**
Search	8	Present full electronic search strategy for at least one database,including any limits used, such that it could berepeated.	**Page.2 and Supplementary Material 1**
Study selection	9	State the process for selecting studies (i.e., screening, eligibility, included in systematic review, and, ifapplicable, included in the meta-analysis).	**Page.5**
Data collection process	10	Describe method of data extraction from reports (e.g., piloted forms, independently, in duplicate) and any processes for obtaining and confirming data frominvestigators.	**Page.3-4**
Data items	11	List and define all variables for which data were sought (e.g., PICOS, funding sources) and any assumptions and simplifications made.	**Page.3-4**
**Geometry of the network**	**S1**	Describe methods used to explore the geometry of the treatment network under study and potential biases related to it.This should include how the evidence base has been graphically summarized for presentation, and what characteristics were compiled and used to describe the evidence base to readers.	**Page.8-9**
Risk of bias within individual studies	12	Describe methods used for assessing risk of bias of individual studies (including specification of whether this was done at the study or outcome level), and how this information is to beused in any data synthesis.	**Page.3**
Summary measures	13	State the principal summary measures (e.g., risk ratio, difference in means). Also describe the use of additional summary measures assessed, such as treatment rankings and surface under the cumulative ranking curve (SUCRA) values,as well as modified approaches used to present summary findings from meta-analyses.	**Page.4**
Planned methods of analysis	14	Describe the methods of handling data and combining results of studies for each network meta-analysis. This should include,but not be limited to:<br>Handling of multi-arm trials;<br>Selection of variance structure;<br>Selection of prior distributions in Bayesian analyses;<br>and Assessment of model fit.	**Page.7-8**

**Assessment of Inconsistency**	**S2**	Describe the statistical methods used to evaluate theagreement of direct and indirect evidence in the treatment network(s) studied. Describe efforts taken to address its presence when found.	**Page.7-8**
Risk of bias across studies	15	Specify any assessment of risk of bias that may affect the cumulative evidence (e.g., publication bias, selective reporting within studies).	**Page.8**
Additional analyses	16	Describe methods of additional analyses if done, indicating which were pre-specified. This may include, but not be limited to, the following:<br>Sensitivity or subgroupanalyses;<br>Meta-regression analyses;<br>Alternative formulations of the treatment network;and<br>Use of alternative prior distributions for Bayesian analyses (if applicable).	**Page.8-10**
**RESULTS**			
Study selection	17	Give numbers of studies screened, assessed for eligibility,and included in the review, with reasons for exclusions at each stage, ideally with a flowdiagram.	**Page.4-6 and Figure.1**
**Presentation of network structure**	**S3**	Provide a network graph of the included studies to enable visualization of the geometry of the treatmentnetwork.	**Figure.2a and Figure 3a**
**Summary of network geometry**	**S4**	Provide a brief overview of characteristics of the treatment network. This may include commentary on the abundance of trials and randomized patients for the different interventions and pairwise comparisons in the network, gaps of evidence in the treatment network, and potential biases reflected by the network structure.	**Page.7 and Figure.2a ,Figure 3a**
Study characteristics	18	For each study, present characteristics for which datawere extracted (e.g., study size, PICOS, follow-up period) and provide the citations.	**Figure.8-9 and Table. 1,Table.2**
Risk of bias within studies	19	Present data on risk of bias of each study and, if available, any outcome level assessment.	**Supplement Figure.1 and Supplement Figure.2**
Results of individual studies	20	For all outcomes considered (benefits or harms),present,for each study: 1) simple summary data for each intervention group, and 2) effect estimates and confidenceintervals.Modified approaches may be needed to deal with information from larger networks.	**Table. 1 and Table.2**

Synthesis of results	21	Present results of each meta-analysis done, including confidence/credible intervals. In larger networks, authors may focus on comparisons versus a particular comparator (e.g.placebo or standard care), with full findings presented in an appendix. League tables and forest plots may be considered to summarize pairwise comparisons. If additional summary measures were explored (such as treatment rankings), these should also be presented.	**Page.8-9 and Figure.2b ,Figure 3b**
**Exploration for inconsistency**	**S5**	Describe results from investigations of inconsistency. This may include such information as measures of model fit to compare consistency and inconsistency models, P values from statistical tests, or summary of inconsistency estimates from different parts of the treatment network.	**Page.7-8**
Risk of bias across studies	22	Present results of any assessment of risk of bias acrossstudies for the evidence base being studied.	**Page.7**
Results of additional analyses	23	Give results of additional analyses, if done(e.g.,sensitivityor subgroup analyses, meta-regression analyses, alternative network geometries studied, alternative choice of prior distributions for Bayesian analyses, and soforth)	**Page.9-11, Figure.4, Figure.5, Supplement SUCRA plot**
**DISCUSSION**			
Summary of evidence	24	Summarize the main findings, including the strength of evidence for each main outcome; consider their relevance to key groups (e.g., healthcare providers, users, and policy-makers).	**Page.11-13**
Limitations	25	Discuss limitations at study and outcome level (e.g., risk of bias), and at review level (e.g., incomplete retrieval of identified research, reporting bias). Comment on the validity of the assumptions, such as transitivity and consistency.Comment on any concerns regarding network geometry (e.g.,avoidance of certain comparisons).	**Page.13**
Conclusions	26	Provide a general interpretation of the results in the contextof other evidence, and implications for future research.	**Page.13**
**FUNDING**			
Funding	27	Describe sources of funding for the systematic review andother support (e.g., supply of data); role of funders for the systematic review. This should also include information regardingwhether funding has been received from manufacturers of treatments in the network and/or whether some of the authors are content experts with professional conflicts of interest that could affect use of treatments in thenetwork.	**None**
