# Supplementary figures and images for: Effectiveness comparisons of various therapies for FIGO stage IB2/IIA2 cervical cancer: a Bayesian network meta-analysis
Source: BMC Cancer. 2021 Oct 6;21:1078. doi: 10.1186/s12885-021-08685-9 (PMC8493709; doi:10.1186/s12885-021-08685-9)

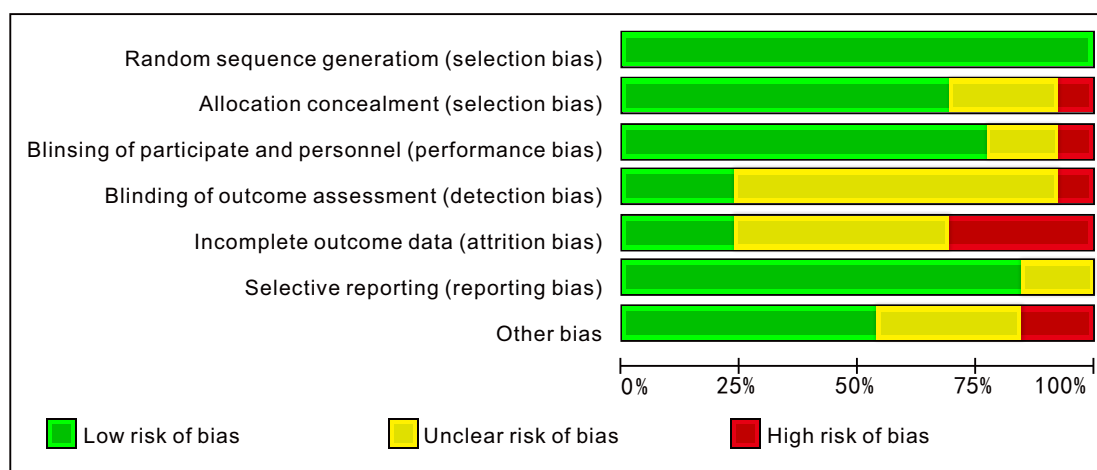

Figure S1. Risk of Bias Graph

Supplement: Supplementary file 3 — Additional file 3. [file 12885_2021_8685_MOESM3_ESM.pdf]

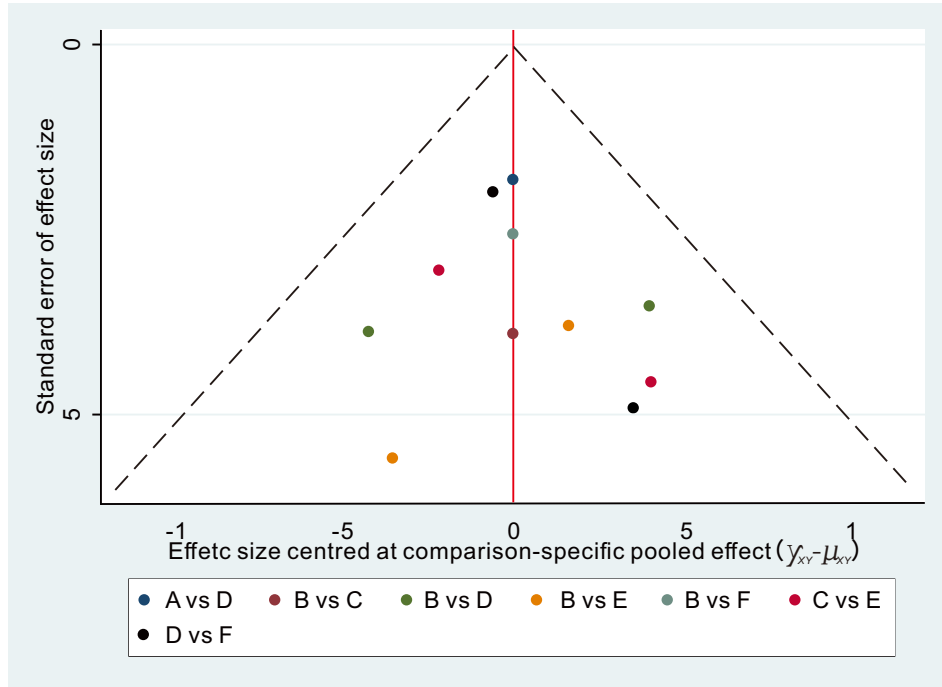

Figure S3. The funnel chart for OS

Supplement: Supplementary file 5 — Additional file 5. [file 12885_2021_8685_MOESM5_ESM.pdf]

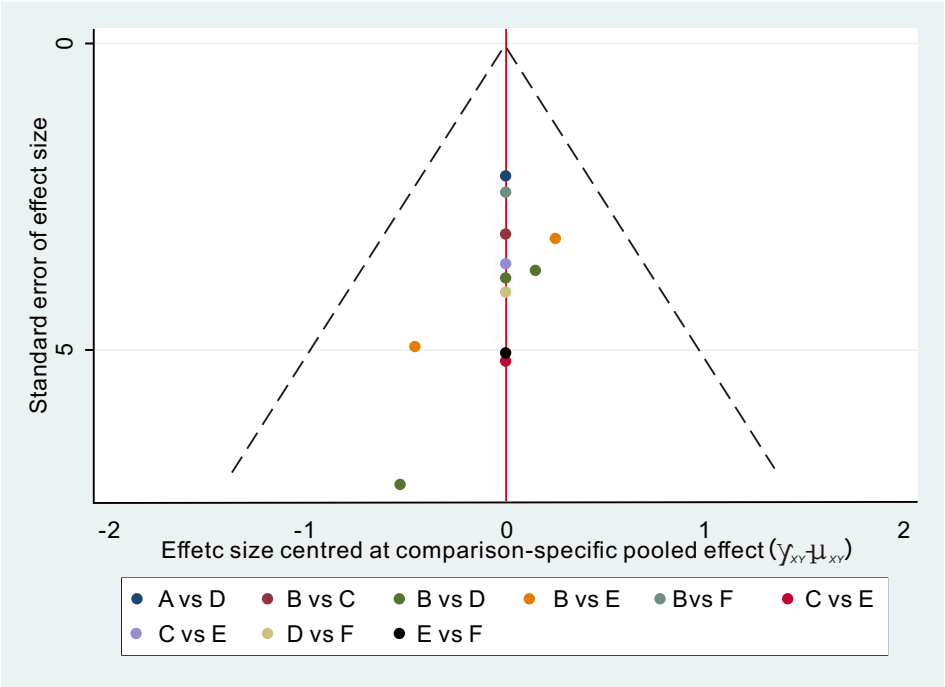

Figure S4. The funnel chart for Relapse

Supplement: Supplementary file 6 — Additional file 6. [file 12885_2021_8685_MOESM6_ESM.pdf]

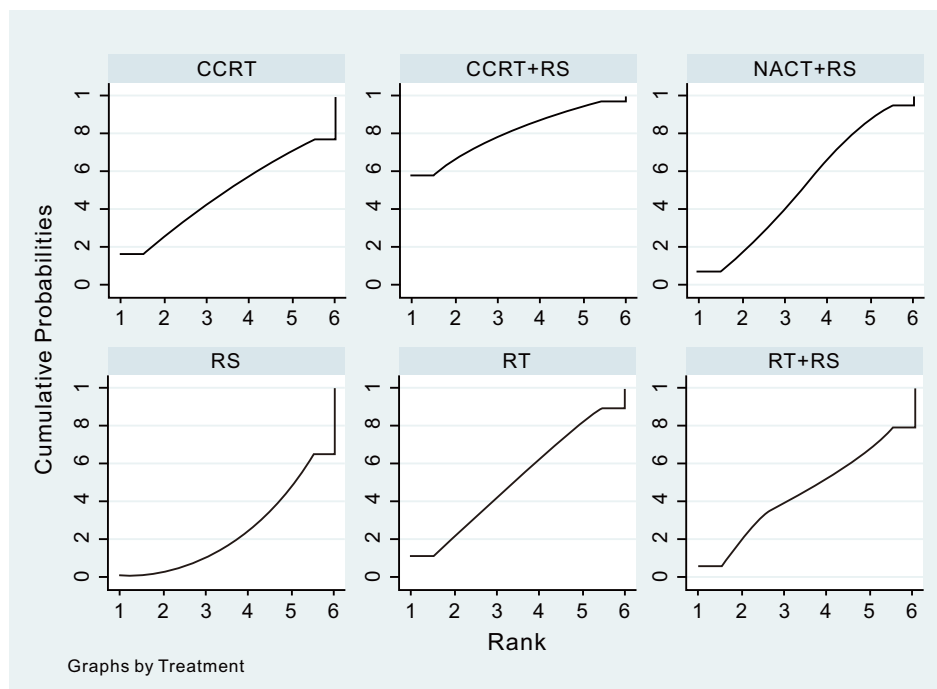

Figure S5. SUCRA Line for OS

Supplement: Supplementary file 7 — Additional file 7. [file 12885_2021_8685_MOESM7_ESM.pdf]

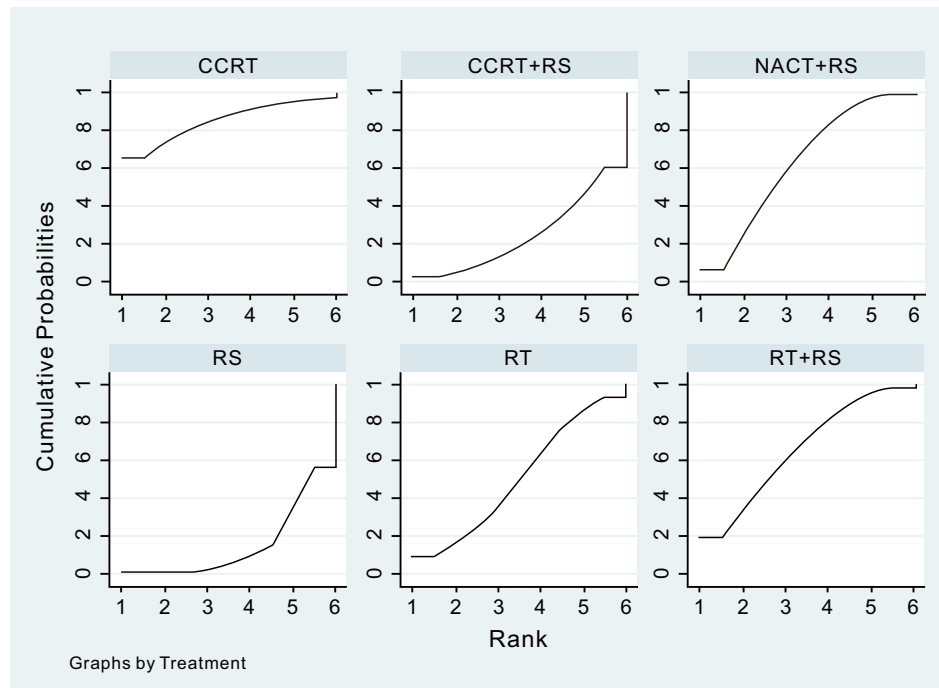

Figure S6. SUCRA Line for Relapse

Supplement: Supplementary file 8 — Additional file 8. [file 12885_2021_8685_MOESM8_ESM.pdf]
