# Supplementary material for: Effectiveness comparisons of various therapies for FIGO stage IB2/IIA2 cervical cancer: a Bayesian network meta-analysis
Source: BMC Cancer. 2021 Oct 6;21:1078. doi: 10.1186/s12885-021-08685-9 (PMC8493709; doi:10.1186/s12885-021-08685-9)
Supplement: Supplementary file 4 — Additional file 4. [file 12885_2021_8685_MOESM4_ESM.pdf]

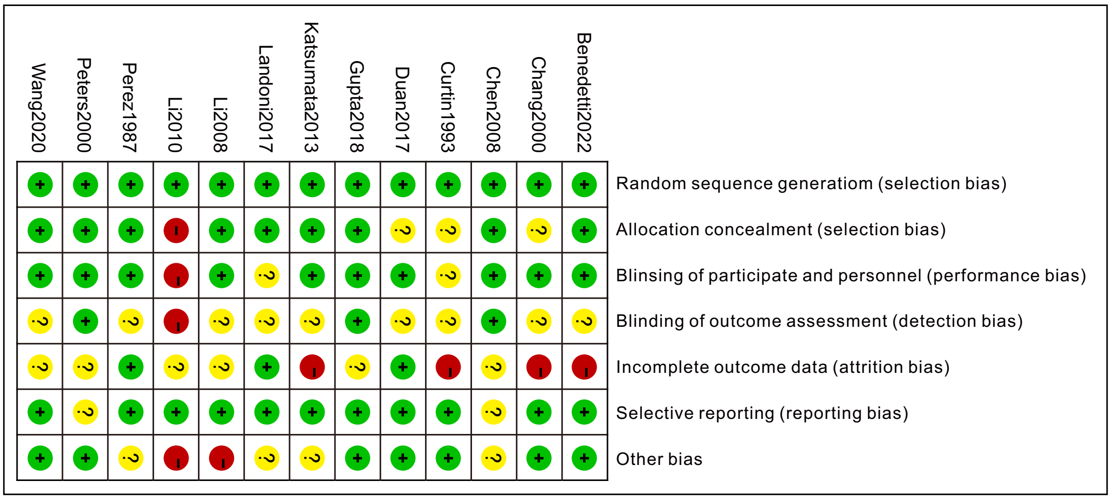

Figure S2. Risk of Bias Summary
