# Supplementary material for: Effectiveness comparisons of various therapies for FIGO stage IB2/IIA2 cervical cancer: a Bayesian network meta-analysis
Source: BMC Cancer. 2021 Oct 6;21:1078. doi: 10.1186/s12885-021-08685-9 (PMC8493709; doi:10.1186/s12885-021-08685-9)
Supplement: Supplementary file 9 — Additional file 9. [file 12885_2021_8685_MOESM9_ESM.pdf]

PICOS	Inclusion criteria	Exclusion criteria
P (Population)	1) Premenopausal or Postmenopausal Women(aged from 18 to 75 years); 2) Women had a diagnosis in stage IB2 or IIA2 Cervical Carcinoma based on FIGO(2009).	Women are undergoing treatment for cervical cancer.
I (Interventions)	1) Concomitant chemoradiotherapy(CCRT); 2) Radical surgery(RS); 3) Radical surgery following chemoradiotherapy(CCRT+RS); 4) Neoadjuvant chemotherapy followed by radical surgery(NACT+RS); 5) Adjuvant radiotherapy followed by Radical surgery(RT+RS); 6)Radiotherapy alone(RT).	the definition of interventions were ambiguous
C (Comparator)	1)Compare each arm to the next; 2)named control group.	control group in any mix with any intervention.
O (Outcome)	1) The overall survival; 2) The relapse;	The related outcome index is not clear.
S (Study design)	Randomized controlled trials has been published without year and language restriction.	1) case control study; 2) cohort study or suppl; 3) full-text but unpublished; 4) Protocol study.
